# The Ohio Lunatic Asylum

**Published:** 1850-10-01

**Authors:** 


					Art. III.-
-THE OHIO LUNATIC ASYLUM.
AVe arc indebted to tlie kindness of Dr. 11. C. Hopkins, late senior
assistant physician to tlie Ohio State Lunatic Asylum, for a series of
valuable reports of this American establishment for the care and cure of
the insane, an analysis of which we now lay before our readers. These
reports are drawn up with great care, and contain a large amount of
Interesting and valuable facts relative to the condition of the insane in
the United States of America. AVe think the physician and medical
officers associated with British Asylums would do well to imitate the
?example set us by our American brethren, and communicate, by means
of their annual reports, something beyond a mere dry detail of the
THE OHIO LUNATIC ASYLUM. 457
yearly expenditure and receipts of the establishments over which they
preside, and the number of patients admitted and discharged during- the
preceding year. These reports ought to contain the results of particular
modes of medical and moral treatment; the details of morbid phenomena
discovered on post-mortem examinations, and a resume of the inte-
resting facts connected with the early and more advanced history of the
many most remarkable cases which obtain annual admission into our
public asylums, illustrative of their origin, treatment, progress, and
termination.
It appears that the Ohio Asylum Avas opened for the reception of
patients in November, 1838. It occupies a beautiful lawn north-east of
the city of Columbus, and is about one mile distant from its public square.
It is a stupendous pile of brick and stone-work, which presents an impos-
ing appearance as seen from almost every part of the State Capital and
surrounding country. The structure faces the south. It is quadrangular,
and measures 37G feet in front, by 218 feet in depth. The buildings
cover just one acre of ground, and inclose an area of 18G4 square yards.
The main centre building is three stories and an attic in height above
the basement, the wings and new buildings are each three stories; and a
walk through all its different passages and galleries exceeds one mile.
The building required 5,200,000 bricks, and very nearly 50,000 feet
of cut stone. The entire cost to the State was about 150,000 dollars,
including the amount of the work done by the convicts of the Ohio
Penitentiary, which constituted a large item in the account.
The principal front displays a handsome columniated facade, orna-
mented with a fine Ionic portico of freestone, from Waverley, in Pike
countv, which was beautifully cut by the convicts. There are four
columns of this stone, each measuring 3 feet 8 inches in diameter at
the base, and 31 feet 2 inches in height. They stand upon a platform
of the same materials, and on a level with the top of the basement,
which is of cut limestone, and 7 feet above the surface of the earth.
The centre building is 95 feet 8 inches in width by 45 feet 6 inches in
depth. The wings recede 25 feet from the front line, and are each
100 feet long in front, and 119 feet 2 inches long in the rear, leaving a
recess behind the centre building, which is 18 feet G inches deep, and
5G feet 9 inches wide. Each wing unites with a superb verandah 44
feet square and 3 stories high, which is built of cut limestone, taken
from the Scioto quarries, 3 miles west of Columbus. Each verandah
exhibits two fronts, supported by four massive piers built of the same
material, cut in the most perfect manner. Between these piers are
stone upstarts which support the windows. The lateral buildings
extend to the north from each verandah, and make right angles with
the wings of the former edifice. They are each 39 feet wide, and 1 / 5
NO. XII. H H
458 THE OHIO LUNATIC ASYLUM.
feet long, exclusive of the verandahs. Two lodges for violent and
filthy patients occupy the rear of the square, each two stories high and
80 feet long, by 18 feet broad. And the area thus inclosed is divided
through its middle by a building of three stories, 114 feet long and 36
feet wide, which is to be connected with the rear of the centrc building
by means of a porch-way of open work, which will be 55 feet long by
14 feet wide, and two stories high.
Counting parlours, kitchens, verandahs, and all apartments with board
floors, and other apartments, the asylum contains 440 rooms, and will
accommodate about 350 patients, together with all the officers, attend-
ants, and assistants, necessary to take charge of them, and carry on the
business of the institution. The buildings are all covered with tin, and
chiefly constructed of brick, except the verandahs, basements, cornices,
and architectural decorations, which are all cut-stone. Every part is
conveniently arranged, and the whole establishment can be well heated,
and is thoroughly ventilated.
When the first edifice was erected, the example of many old institu-
tions was followed in providing court-yards with high fences for the use
of the patients, that they might enjoy the benefit of exercise in the open
air. But experience soon demonstrated the uselessness of this arrange-
ment in a climate of so much variableness as the American, in which
but few days occur in the year when patients could safely be permitted
to lie about upon the ground?a habit to which many lunatics incline,
and which they almost always practise in these yards. In supplying their
place, the example of the State Hospital at Worcester, in Massachusetts,
was followed, by erecting verandahs at the corners for exercise and
recreations, and there has not been the least reason to be dissatisfied
with the change. The verandahs arc always ready, in all seasons, and
in all kinds of weather; and arc more cheerful and less forbidding than
court-yards. They are also dry, neat, and clean, so that patients can
follow out their inclinations in comfort, and without exposure.
Every gallery in cacli building occupied by the patients opens into
one of these delightful verandahs, which is of improved construction,
and 37 feet square inside. They have beautiful floors, Avith conducting
gutters along the margin, to carry off all water that may be used in
cleaning, or that may blow in from rain; and their ample windows be-
tween the piers, and upstarts, of cut-stone, arc finely arranged with fixed
cast-iron sash for security, and moveable glazed wooden sashes for ventila-
tion, or protection during inclement weather. These wooden sashes can
all be pushed up behind the immense stone panels which connect the
piers, where they are entirely out of sight. This admits the fresh air in
any desirable quantity, and all being elevated so as to command an exten-
sive view of the city and neighbouring country, they afford places of much
?i
EARLY LEGISLATION ON THE SUBJECT OF INSANITY. 450
delight to the patients, and are constantly resorted to by all classes
during a very large portion of the year.
The earliest legislation upon the subject of idiots and lunatics, in
Ohio, was an act of the territorial governor and judges, passed August 1,
1792, four years after the organization of the territorial, and ten years
before the organization of the state government. It provided for the
trial of the question of insanity by a jury, and the appointment of
guardians, with plenary powers, over the estates and persons of the
insane, but contained no provision with reference to their safe keeping,
or remedial treatment. Paupers were required to be provided for, in
the same manner as other poor. No further legislation upon the
subject, worthy of mention, occurred until the passing of the act of
February 13, 1815, entitled "an act to provide for the safe keeping of
idiots and lunatics, and for other purposes." That act re-enacted all
the provisions of the preceding acts, and provided, further, that the
jury should certify, in addition to their other finding?"whether such
person ought to be put into close confinement." If their verdict upon
this point was in the affirmative, the lunatic was required to be com-
mitted to the county jail, unless his friends should give sufficient bond
for his safe keeping. If committed to jail, the county commissioners
were required "carefully to examine him, and if, in tlieir opinion,
medical aid should be requisite, to employ a skilful physician." If it
were not necessary to confine the lunatic, and he were a pauper, he was
required to be supported by the ovei'seers of the poor, in the same
manner as was provided by the pre-existing acts.
These provisions continued in force, without any material modifica-
tion, until the passing of the act of March 9, 1838.
Such was the state of the American law upon this subject, when the
Ohio institution came into operation, and the act of 1838, above men-
tioned, took effect.
Prior to that time there was no private or public institution in the
state where the insane could be received and properly treated. A few
patients were confined in the hospital at Cincinnati; but the space that
could be allotted to them was too limited, and the other accommodations
were too unsuited to their condition to admit of the possibility of proper
curative treatment. The inmates of that institution are understood to
have fared no better than those confined elsewhere, in the county jails.
The fate of the insane, throughout the state, was hard, indeed. But
little attention had been paid, by the medical profession, to this class of
diseases, and but few had made themselves acquainted with the proper
mode of treating them. A few of the insane were sent abroad, to
institutions in other states. Many of them wandered at large with all
the melancholy marks of their blight thick upon them. Others were
h ii 2
460 ORIGIN OF THE OHIO LUNATIC ASYLUM.
watched over, or confined by tlieir friends, with hopeless anxiety, while
the more dangerous class glared through the iron gratings of the
county jails. Little or nothing was done for them?whilst every hour
the facilities of cure were diminishing, and their diseases were growing
worse.
The number of insane in the state, in 1835, who were deemed fit
subjects for an asylum, (exclusive, of course, of idiots, and those incur-
ably affected,) were estimated, upon reliable data, at upwards of two
hundred. According to the returns made to the governor in that year,
there were two hundred and ninety-four supported at the public expense,
ninety-four of whom were in confinement.
Thick darkness, relieved by no ray of light, seemed to enshroud the
entire class.
Such was the state of things when the attention of the public was
called to the design of this institution. Its germ ivas planted by the
medical convention of Ohio. That body, at its first session, in January,
1835, adopted an eloquent memorial to the legislature, recommending
the establishment of such an institution. The memorial was presented
to the General Assembly then in session. It touched a chord that
vibrated at once, with keen sensibility, throughout the state. The
legislature yielded promptly to the generous impulse from without, and
at the same session passed an act, making an appropriation for the
purchase of a site, and providing for the appointment of trustees, and
the erection of the edifice. The trustees visited the leading institutions
in the eastern states, and immediately upon their return, purchased the
present site. They .also made an elaborate report to the legislature, at
its next session, detailing the results of their observations, and the plan
of the edifice they had fixed upon. A libcx*al appropriation by the
legislature followed. The work was vigorously commenced. The
directors of the penitentiary appropriated a large share of convict
labour to it. Other appropriations, by the legislature, were made, and
the work proceeded rapidly.
In March, 1838, the legislature passed "An Act to provide for the
Government ot the Ohio Lunatic Asylum"?and, in the month of
November following, the institution was organized, and came into
operation under the care of the present efficient and valuable superin-
tendent.
The administration of the institution is conducted by a superin-
tendent, (who is its medical head, and has the entire care and super-
vision of all its departments,) an assistant physician, a steward, a
matron, and the proper number of attendants, and other subordinates.
The usual annual appropriation for the support of the institution is
about 13,000 dollars. A further sum of about 3000 dollars is received
ADMISSION OF PATIENTS. 461
annually from pay patients, making an aggregate of about 1G,000
dollars. The sum covers all expenses.
The laws in force, at present, in regard to idiots and lunatics, like
all the preceding acts, require the,fact of insanity to be established by
the verdict of a jury. The jury is required to set forth whether the
person complained against " is an idiot, that is to say, a person naturally
without mind"or lunatic;" "whether, in their opinion, he is so
furiously mad as to render it dangerous to the peace and safety of the
community that he should be permitted to go at large ?" whether he
is a pauper, or has any estate, and the supposed value, and annual
income of such estate"?and, also, whether the pauper has been a resi-
dent citizen of the state a year. When it is desired to procure the
admission of a pauper, who has been in a state of mental derangement
for a longer period than two years, "it shall be clearly proven that it
would be dangerous to the peace and safety of the community for him
to go at largeand this fact, also, must be stated in the verdict of the
jury. The provisions in regard to the support and persons of the class
not entitled to admission, and to the management of the estates of the
insane, are substantially the same as those formerly in force. Where
" such person is a lunatic" and a pauper, and has been a resident citizen
of the state a year, and his disease is of not more than two years dura-
tion, or, in that event, he is so furiously mad as to be dangerous, it is
required that a warrant shall be issued for his commitment to the asylum.
If the person be not a pauper, the warrant is not required to issue,
unless it be demanded by those at whose instance the trial was had.
Upon the issuing of the warrant, if the person be a pauper, or furiously
mad, the sheriff is required (unless bond be given for his safe keeping)
to commit him to the poor house of the county, if there be one, (unless
he be specially directed to commit him to the jail,) and if there be no
poor house, then to the jail, and to make immediate application for his
admission into the asylum. If lie cannot then be received, notice is to
be given to an associate judge, if the person be a pauper, and if other-
wise, to his guardian. The like notice is to be given by the sheriff,
where a lunatic has been discharged from the asylum " on account of the
incurable nature of his malady." If the person be not " furiously mad,"
lie is to be disposed of in the same manner as those not entitled to
admission. If he be "furiously madthe associate judge is required
(unless a sufficient bond be given for his safe keeping) to issue a warrant
for his commitment; whereupon, he is to be committed, as is required
in the first instance. "A skilful physician" is to be named in the
warrant, to attend him, and he is to be thus kept until received into the
asylum, discharged by order of the physician, or otherwise provided for,
by the county commissioners.
462 ADMISSION OF PATIENTS.
No "idiot or person, naturally without mind," can be received into
the asylum. And those who arc found to be incurable are required to
be removed to it, unless tliey shall be " so furiously mad as to render it,
manifestly, dangerous to the peace and safety of the community that
they should go at large." "Pay patients" may be received "upon the
certificate of two respectable physicians," when there is room for them;
and " if, at any time, it shall be certified, by the proper authority, that
the estate of such person is insufficient for his or her support in the
institution, after deducting from such estate the amount neccssary for
the maintenance of the family of such person, such person shall be
supported in and at the expense of the institution."
When the asylum is full, the superintendent is required "to keep a
record of the name and date of each applicant," and whenever a vacancy
shall occur, "he shall cause notice to be given to the clerk of the Court
of Common Pleas, of the proper county, that the first named on the list
of applicants in the county having the least number under the charge
of the institution, in proportion to the population, will be received,
provided that, in all cases, preference shall be given to paupers and
citizens of the State of Ohio."
This asylum was built at the expense of the state, and chiefly by the
labour of convicts from the Penitentiary. It will be interesting to our
medical friends connected with British County Asylums to be made
acquainted with the mode of admitting pauper patients into the
American public institutions. In many respects it differs essentially
from the practice followed in our own country. We would, however, pre-
mise that each American state has its own peculiar mode of procedure
in these cases.
When it is clearly ascertained that an individual is deranged in
mind, and that it is expedient that he or she should be committed to a
lunatic asylum, the first step is to make application in writing to one of
the associate judges of the county in which such person resides. '
This is done in the following manner:?
"March, 18 .
"To the Honourable
" Sir:
" This application will represent that A. B., of
a resident citizen of the State of Ohio, is in a state of mental derange-
ment. His circumstances require that he should be supported at the
public expense, and I respectfully ask that his case may be inquired
into according to law. Respectfully, &c."
The associate judge thereupon issues his warrant, as directed by the
statute, and it becomes necessary that the lunatic should appear before
Iiiin at the county seat, on the particular day named. This warrant is-
K ADMISSION OF PATIENTS. 463
directed to the sheriff, but it may be executed by any other person, and
as the presence of the sheriff, or any known executive officer of the
county, may give offence to the poor lunatic, who is wholly unconscious
of crime, or of having done anything that is wrong, it is considered best
k for one or two friends to attend to this duty, and in most instances they
are enabled to persuade the deranged person to accompany them in a
> . pleasant, sociable, and friendly manner, without the necessity of using
force or violence of any kind.
The lunatic is neither told that he is thought insane, nor that he is
in custody and about to have a trial; and on arriving at the county
seat, he is taken immediately to a private room, where the judge and
physicians see him; also the jurors, if necessary. He is never allowed
to appear before the jury so as to witness the proceedings, or hear any
of the testimony that may be given in his case.
The jury consider the following points: 1. As to the fact of insanity
and its general character as to violence. 2. The duration of the disease.
3. Whether the person is a pauper; and 4. The question of citizenship.
If they are satisfied on these points, and are of the opinion that such per-
son is entitled to admission into the lunatic asylum according to the pro-
visions of the statute, their verdict is drawn in the following manner:?
"We, the jury empannelled and sworn to inquire into the case of A.B.,
of do find that he [or she] is insane. The disease
appears to have existed for about the period of months,
[or years,] and in our judgment it has been clearly proven that it would
be dangerous to the peace and safety of the community for him [or her]
to "'0 at large. We are also satisfied that he [or she] is a pauper, and it
has been established by legal and proper testimony before us that the
said A. B. has been a resident citizen of the state of Ohio for the period
of one year next immediately preceding the date of the application.
Witness our hands the day of
OR,
" We, the jury empannelled and sworn to inquire into the case of A.B.,
0f do find that he [or she] is insane. It has not
been clearly proven that it would be dangerous to the peace and safety
of community for him [or her] to go at large, but the mental derange-
ment has not existed for a longer period than two years. We are also
satisfied that he [or she] is a pauper, and it has been established by
legal and proper testimony before us that the said A. B. has been a resi-
dent citizen of the state of Ohio for the period of one year next immedi-
ately preceding the date of the application. Witness our hands the
day of
After the verdict of the jury is received the judge issues his warrant
for the commitment of the insane persons to the lunatic asyluim This
warrant is directed to the sheriff of the county, who makes application,
464. MORAL TREATMENT OF THE INSANE.
in -writing to the superintendent of the asylum to ascertain whether such
person can be received. " In which application he sets forth the name,
age, sex, arid place of residence of such person, together with a copy of the
verdict of the jury of inquest," as required in the 4th section of the act,
" providing for the safe keeping of Idiots, Lunatics," &c.
Particular attention is said to be paid, in this asylum, to the moral
management of the patients, always recognising the great principle of
treatment to be embraced in the single idea, humanity, the law of
love?that sympathy which appropriates another s consciousness of
pain, and makes it a personal relief from suffering, when another's
sufferings are relieved .*
The moral government is kind and respectful, and, as far as pos-
sible, parental; with a becoming firmness at all times in respect to the
order and discipline which reason and experience have approved. As
a primary step, the officers are anxious to secure the confidence and
good-will of the patients, and endeavour to retain it, by kind hospitality
and attention to their wants, without any extraordinary officiousness or
unbecoming authority; always desirous that they shall have every
allowable privilege, and participate in the enjoyment of every pleasure
which their capacity and condition may admit. Males and females, in
separate parties, frequently ramble over the country in all directions,
accompanied only by a single attendant. The matron regularly invites
all the ladies, who behave well, to her social parties, on Thursday after-
noon : and a carriage is always ready, in pleasant weather, if they feel
anxious to take a ride. The gentlemen are provided with a variety of
amusing games, and books and stationery arc freely distributed to all.
There are sports on the green, and music and dancing parties in the
halls, and the National Independence on the 4th of July, is also, in
obedience to common custom, duly celebrated.
The system of moral discipline docs not depend upon either secret
arts or physical force. It is entirely based upon the plainest and most
simple principles of parental kindness and common sense, with such tact
and ingenuity as necessity may suggest, or occasion require. " A cheerful,
encouraging, friendly address ; kind, but firm manners ; to be patient
to hear, but cautiously prudent in answering; never making a promise
that cannot safely be performed, and, when made, never to break it;
to be vigilant and decided; prompt to control, when necessary, and
willing but cautious in removing it, when once imposed;"?these are
qualities which will command the respect, and gratitude, and attention of
the misguided lunatic, when they could never be otherwise attained.
Skill is superior to force; steadiness and firmness are infinitely
to be preferred to rashness and violence; and a well turned joke often
* Report of Worcester Asylum, Massachusetts.
HOMICIDAL INSANITY. 465
succeeds where other means fail. The great points are a hind heart,
pure motives, and sound judgment, directed by a knowledge of the
habits and wants of the insane.
In the Second Annual Report, we find the following interesting par-
ticulars relative to homicidal insanity. It is observed
" This is a form of mental derangement in which there are few or no
delusions, or hallucinations, and apparently very little impairment of
the intellectual faculties; but a morbid condition and perversion of the
moral sense and social affections, feelings, and propensities. Persons
thus diseased may be capable of reasoning, particularly when calm ; but
under any excitement, the moral alienation entirely misleads and per-
verts the judgment, and the consequences can neither be estimated nor
regarded. Directed by an impulse, which is headlong and irresistible,
the unfortunate subject of this disease may be hurried to deeds of out-
rage and blood, which will shock the stoutest hearts, and excite the
strongest sympathies of human nature. "We know that it is exceedingly
difficult effectually to distinguish between vice and perverseness, and
the legitimate consequences of disease; but certainly there is such a
distinction, and it is clearly marked by the following strong and
remarkable features: want of motive ; unconsciousness and indif-
ference AS TO THE CRIME; ADMISSION OF TIIE FACT, WITH THE ABSENCE
OF GRIEF, REMORSE, REPENTANCE, OR SATISFACTION.
" To illustrate this important definition, we give the history of this
case, with all the important circumstances in detail, as taken down
from the simple narrative of the person who is the subject of this
peculiar form of disease, now entirely free of excitement, and appa-
rently in the enjoyment of reason, at least upon all the ordinary
matters of life?a modest and diffident female, with a pensive and
imploring countenance, and perhaps the last person that would be
selected as a homicide in our halls. Her body is now wasting to the
tomb, and her spirit must ere long ascend to God, who gave it.
" < I was born in the state of Maryland, and am forty-four years of age.
From my earliest recollection, I was of a quiet and steady turn of mind,
and have seen nothing but hardship and trouble all my days. I was
married in my twenty-fourth year, in opposition to the will of my
parents, but was devotedly attached to the man of my choice. He
received an injury in his shoulder some time after our marriage, and I
was in the habit of assisting him with his work 011 the farm. I worked
uncommonly hard at making fence, burning brush, and clearing up the
land. The stooping, heat of the sun, and hot fires of the burning
brush, appeared to affect my head very much. On a certain day, while
engaged in the field, I was suddenly struck almost blind, and felt an
uncommon stiffness in the back of my neck, accompanied with a
drawing down of the skin over my eyes and forehead, and the sensation
of tight cords passing through my head. It was some time befoie I
felt able to return to the house, and attend to my domestic duties. I
had lost much sleep for two or three weeks previous to this attack, and
felt troubled in my mind 011 account of our difficulties in getting along
4G6 CONFESSION OF A HOMICIDAL INSANE PATIENT.
in tlie world. On the following night I was greatly distressed, and
thought somebody was coming to kill me. I could not go to sleep,
and, by morning, I believe I was completely deranged. I continued
out of my head for three or four months, and suffered much distress and
anxiety of mind, from the apprehension that I was to be killed; but
through the attention of the physicians, and kindness of my husband, I
began to recover by degrees, and eventually got entirely well.
"' After I got well, we concluded to come out to the state of Ohio.
We were very poor, and the journey was accomplished on foot. It was
in March, and the three children and myself suffered greatly from cold
and fatigue. Husband had taken to drink, and we had hard work to
get along j and in the month of November following, I had another
attack of derangement. I forgot to tell you, that my health began to
fail previous to my first attack (Amenorrlicea and Leucorrhcea), and I
think this brought on the second attack also. I continued ill for several
months, during which time we removed to the north-west part of the
state. I did not know what was to become of me, my distress was so
great, that I longed to make my escape, and hide where no mortal
could find me. "VYe again had to make our journey on foot, and I cried
and fretted most of the road. I wished I never bad been born, and
often said to my husband, 1 There's my poor children, and I've got to
go hell for having them he would scold me for talking so, but I could
not help it, such dreadful thoughts would come into my head, in spite
of all I could do. I sometimes tried to drive it out of my head, by
beating it against the fence. Frequently it appeared to my mind
as if it would rain hail and fire upon my head, and I should be beaten
to pieces with thunder and lightning ; and when I did, once in a great
while, fall into a troubled sleep, I would suddenly start up in a fright,
with my hands before my face, to keep the awful danger off. It was all,
however, respecting myself and the children; I did not think that
anything was to happen to their father.
" ' At this time, husband was sometimes a little crabbed, but he could
not get any liquor in them parts, and did not get drunk. I was as
much attached to him as ever, for he was a kind and good man to me.
I don't think two persons could be fonder of each other. At last, how-
ever, I took it into my poor head that he was going to kill me ! This
painful idea continued to torment my mind for two or three weeks. It
was dreadful. We had lived together so many years, and why should
he want to kill poor me 1
" 1 One Sunday, I was full of this idea the whole morning, and about
twelve o'clock, ran off on to the wild prairie, where I wandered about
during the whole afternoon, and did not think of returning until near
night. I met husband coming after me, with one of the children, and
Ave all returned to the house together. I got the supper, and the
family went to bed, as usual. I could not sleep. It was a terrible
night to me. About day-break, I got up and built a fire. Something
appeared to tell me there was dreadful work to be done. I was vcry
much agitated when the thought came into my head that I must kill
him; but my mind was so much excited, I cannot tell anybody exactly
how I felt. The same thought came into my head in the night, but I
CONFESSION OF A HOMICIDAL INSANE PATIENT. 4G7
succeeded in putting it down. I had a confused notion that I was
horn to he lost; it appeared like a hidden mystery; hut the thought
that I was horn to he lost was uppermost. At the same time, I sup-
posed he would he saved. I often thought that everybody was made
righteous beside myself.
"f I stood alone by the fire. All were sound asleep. Husband partly
wakened when I first got out of bed; he merely opened his eyes, and
then went to sleep again immediately. I knew he was sound asleep,
and I felt that I must kill him to save myself. I accordingly went to
where the children lay, and' drew out a broad axe from under their bed,
that he had borrowed from a neighbour. I went right to his bed, with
the axe in my hand, trembling like a leaf. He was laying on his right
side, with his neck bare, and I immediately struck him the one fatal
lick across his neck ! He kind o' struggled, and partly raised himself
to his knees, and wakened the children, a dying. My daughter came
running to me in a fright, and took the axe out of my hands, screaming
that I had murdered father ! and sprang to him, and kissed him on his
forehead, crying, ' Oh ! he's my poor, poor father !'
" ' As quick as they could get their clothes on, the children ran off to
the neighbours. I sat down, and stayed in the house alone, until the
neighbours came. A gentleman first looked in at the door, and asked
me what I had done. I said (evasively) that I had not done anything;
that I had to go to hell, and that I would have something to go there
for. He came in, and said, he must tie me. I told him, I did not
want to run away, and would go along with him without tying. He
first took me to the next house, and in three days they sent me to jail.
I was as distracted as ever; and what I had done gave me no relief nor
satisfaction. I think it was as much as three months before I began to
come to myself. I was not tried for the murder, which I never
attempted to deny, but sent here to the lunatic asylum. I supposed
they would hang me, and did not expect anything else for a long time.
My mind now appears to he entirely clear, and I want to go home to
my children. I feel much better, though very weak. I am thankful
they brought me here. My mind is altered now about going' to hell; I
have hopes, and think, when I die, I will go to rest. I like to go
to your evening worship very much, when I am able to walk up
stairs,' &c.
"To our question, 'Well, Mrs. S., you say your mind is now clear;
don't you know it was wrong for you to kill your husband V 'Yes,
doctor* I know it was wrong.' ' And are you sorry that you did it V
This question appeared to touch the very cord that had been so long
diseased. Her eyes flashed; the pupils contracted; and her whole
frame shook, as she raised herself up, and emphatically replied, ' No,
doctor, 110 ! I'm not sorry for it! It was God's will?why should I he
sorry? He made me do it, to show me His power?and I was willing
to do something to go to hell for !' It was but the flash of a moment,
and all was calm as before. Her next remark was in reference to her
general health, and perfectly rational."
Wc are glad to perceive that the importance of daily religious exercises
408 RELIGIOUS EXERCISES IN LUNATIC ASYLUMS.
meets with a just appreciation 011 the part of the conductors of this great
institution. It should never be forgotten that in asylums those who are
entrusted with the responsibility of its government have an extensive
family to govern, as well as to provide for the daily wants of the insane;
and, merely as a moral regulation, the daily religious service is of the
first consequence to inspire confidence and respect, and to harmonize the
feelings; hut above all this, says the writer of the report before us, " we
are fully prepared to add our testimony to the importance and value of
religious exercises with the insane, as a special mean of grace, well
calculated to bring light and wisdom to the mind, relief and peace to
the heart and conscience. By many of the convalescent, it is estimated
as a blessed privilege; and in respect to them, there can be no doubt of
its propriety. We have never seen any evil effects from the practice,
with the more diseased and unsteady; and when we find many of the
positively deranged who are anxious to accompany the rest, and spend
half an hour in social worship, and not only conduct themselves with
propriety and order, but sincerely thank us for the privilege, Ave are
satisfied, that in many instances the soul has been refreshed, though
there may have been but an imperfect and beclouded view of a merciful
Redeemer." The report continues,?
" When the bell rings for worship, the patients gather in from the
different galleries, each several class accompanied by its proper attendant,
and all are regularly seated, without strife or confusion; males on the
left, and females 011 the right of the superintendent's table; and there is
the most respectful attention when the blessed Bible is opened, and the
Avords of eternal life arc read. There, amid the group upon the right,
is the countenance of one Avho has beenAvitness to many sorroAVS. Upon
the opposite side, sparkle the Avild eyes of a stronger mind in ruins; and
directly in front, is the pale form of the female homicide, Avho, Avith one
awful stroke, severed the head from the body of her sleeping husband!?
all ready and Avilling to unite in the praise of God, and, in greater or
less degree, enjoy the spiritual blessings of the gospel of righteousness
and peace.
" In the Avreck of mind and loss of reason, perhaps the very last ideas
Avhich remain Avill be something of the reverence and respect Avliich is
due to the ' Maker of our frame;' and it is not at all uncommon to see
the aged and demented Christian reverently bend the knees, Avhen years
of darkness, to all human appearance, have shut out all correct know-
ledge of earth and heaven."
We quite concur in the opinion expressed in the report, that there can
scarcely be too much caution exercised in visiting or Avriting to those
Avho are suffering under a disease of the mind. From painful experience,
avc should select the imprudent and unseasonable presence of a near and
dear friend, as the very hardest trial to Avhich an insane mind could be
HOMICIDAL INSANITY. 409
exposed: and long and tender letters containing some ill-timed news, or
the melancholy tidings of sickness and death in the family, may destroy
?weeks and months of favourable progress. As a common rule, letters
to the insane should be short and encouraging, and the county news-
paper, with the name of the friend Avritten on the margin, in many
instances, will be a most valuable substitute.
As reason dawns upon the disordered mind, and the convalescent
state becomes apparent, an intense anxiety is often felt to hear from
"home, sweet home!" with all its endearing and tender associations; the
mind is then impatient of further restraint, and unwilling to submit to
the probationary process which is necessary to complete and establish
perfect recovery: this imposes a very important and painful duty upon
the superintendent; and it is under these circumstances that a prudent
forbearance on the part of relations and friends, is essentially necessary,
desirable, and useful.
The practice of liberating homicidal patients, after acquittal on the
plea of insanity, is severely censured. Dr. Woodward justly observes?
" That all homicides should be perpetually confined. No argument
should weigh, for a moment, with a court of justice, in favour of
liberating such an individual. The fact, that life has been taken, should
overbalance all motives to send such a person into society again, while
the delusions and estrangements of insanity continue; and, we add, not
until months, if not years, of peace, and freedom from excitement,
shall have confirmed their entire release from this dangerous form of
disease.
" We recently attended (says the writer of the Report), an interesting
trial on a subject of this nature in a neighbouring county of this state.*
An habitually peaceful and worthy man was indicted for the most shock-
in^ murder of his wife, with an axe, and a horrible attempt upon the
lives of his children with the same weapon. The facts were not denied,
and his only defence was, that of insanity. He was acquitted, prin-
cipallv upon our testimony as to the fact of his being insane at the time
the murder was committed, of which we have not the slightest doubt;
but our astonishment was only exceeded by our alarm, when subse-
quently informed that bail had been admitted, and this afflicted, but
truly dangerous man, was permitted to go at large. _ This ought not to
be so Science and humanity may interpose for the life of the homicide,
but society should for ever be protected from the effects of his dreadful
disease The lunatic asylum is their proper place; and it should be
duly prepared for their reception and detention."
The particulars of the following inveterate suicidal case will be read
with interest:?
The patient had been insane some months previous to his admis-
sion, all of which time had been spent in the solitude and gloom of a
* Oliio.
470 SUICIDAL INSANITY.
county jail. When brought to the asylum, his condition was truly
deplorable. A fixed and cheerless melancholy, unvisited by a single
hope, had settled down on his mind, making existence so dreadful that
but one thought and one desire seemed to possess him, and that the
vain wish to drop the ills he had and fly to worse, by terminating his
own existence.
Medical treatment was long persevered in with but little encourage-
ment. The suicidal tendency remained strong ; and several unsuccessful
attempts were made to accomplish his purpose. At one time, he
suddenly jumped up from the table, seized a knife, and fled to the water
closet, where the attendant, who immediately pursued, found him, with
a commendable care of a clean floor, leaning over a tub, and vainly
endeavouring to cut his throat with the dull blade. Finding the tool
insufficient, he surrendered it, requesting one sharper, as that would not
answer his purpose. On another occasion, impelled by the same blind
impulse, he succeeded in dropping himself down the large iron pipe
by means of which the gallery is heated. Having remained in this
uneasy, confined posture for some time before he was discovered, he
was drawn out with some difficulty, and in rather an amusing style.
Fortunately, it was at a season when there was 110 fire in the furnace.
When released from this disagreeable confinement, his only feeling
seemed to be that of regret that his efforts were of so little avail.
By a faithful perseverance in medical treatment;?with a constant
appliance of moral means, some little improvement was secured.
Inducements to labour were held out, and this best of all moral means
had the happiest effects. Suddenly he seemed to wake, as from a
dream, happy to find that the fearful delusions of the past were not
realities. With a full realization of the change, he rapidly improved,
and soon left the Ohio Asylum entirely well.
Nothing can give to the physician of an asylum for the treatment of
the insane a higher amount of gratification and honourable pride, than
the fact that those Avho have been confined under his care on the ground
of insanity, and over whom lie has been compelled to exercise a strict
surveillance during the period of their illness, retain, after their recovery
and removal from the institution, kindly feelings and recollections
towards those whose duty it was to interfere thus with their free
agency. Most medical men associated with institutions of this kind,
at times receive letters from the patients Avho have left the asylum
cured, expressive of the most sincere thankfulness for the inestimable
benefits they have received during their temporary confinement and
seclusion from society.
Illustrative of this point, wc copy the subjoined letters, published in
the Third Annual Report. It is unnecessary to observe, that the letters
are from patients who were confined in the asylum:?
CONFESSIONS OF THE INSANE AFTER RECOVERY. 471
? October 12tl), 1841.
" My Dear Sir,?I am now engaged in writing to some of my
friends at the asylum; and though you may not be expecting a letter
from me, yet I must ever consider myself under obligations to you, as
tlie instrument, in tlie hands of Providence, in restoring me to health,
reason, and my family. Of course you do not rank least in my affec-
tions, when I remember my friends at that truly benevolent institution,
for I am fully confident, that had I not been placed there, I should
never have recovered from the torments of a deranged mind.
" Should I undertake to describe to you the anguish which I suffered
before, and for several weeks after I became your patient, my language
would fall so far short, that I should convey no idea of it; but in our
hall I found those that were under the same delusions that I was.
One would say her children were murdered, and she had eaten them.
Another would say, she was to be burned alive, and she was brought
there to be boiled, and the doctors were to make an anatomy of her, &c.
All these, together with hundreds more of the most horrid delusions
that can possibly enter the imagination of the crazed brain, had haunted
me for months. My brother, my husband, and even my own son,
a child of ten years, I was afraid of. I thought everybody on earth
knew my thoughts, and that I was not a human being; that I was the
devil! and that I ought to kill myself and children. I once told
my husband I would kill my boy, for he had already been murdered,
and he was only the ghost of my child. The poor boy cried, and came
to me, and said, 'Yes, mother, I am your boy;' so I could not do it
then; but myself I was fully determined to murder, before I got to
the asylum; for I believed the people of   had called a meeting
on my account, and had resolved to send me to Columbus to be burned,
and made an anatomy of; but when I found others in the asylum, who
seemed to suffer in a degree the same fears and torments as myself, I
was led to try to think I might be wrong in some things, until gradually
reason returned, and with it the affections of the heart.
" When I entered the asylum, my sufferings cannot be described;
and though I do not believe that any being on earth ever suffered any-
thing to be compared with my anguish and torments, yet, if persons
who are deranged do suffer even a thousandth part as much as I did, I
am sure I pity them from my very soul.
" I learn that there are now many applications from persons who
cannot get their friends admitted to the privileges of the institution for
want of room. This, my dear sir, I am certain is not as it should be,
could the people of the beautiful state of Ohio be awakened to the
importance of the subject! Could they but feel for one moment what I
endured for months after, my husband and friends tell me, application
had been made for admittance, I do believe it would be the first subject
they would take into consideration, and their delegates would receive
instruction to extend the institution to whatever distance might be
necessary to admit every sufferer in the state.
" I arrived safe home, and found my children and friends well, and not
a little astonished to see me so soon?and so well, too ; I could scarcely
472 CONFESSIONS of' the insane.
make them know me. Before I left them, and since last February, I
scarcely ever spoke to any one of them, and they seem surprised to hear
me tell how much I suffered; and they wonder when I try to convey to
them some faint idea of the many awful and horrid delusions I was
under. What a dreadful thing it is to have had my children afraid of
me! Now they are so happy, and say, ' Now, mother, ice will keep
house good the next time we try? and they tell the neighbours, 'My
mother has come home, and she is not crazy at all.'
" I think of visiting you all this fall, tfcc. Give my love to all my
female acquaintances, and also your children, little Mary, John, and
Woodward. Yours, respectfully," &c.
"January 21st, 1841.
" Dear Sir,?As you desired me to give you some account of the
manner in which 1 was taken sick, and the circumstances attending my
long affliction, I will now endeavour to state them as near as my recol-
lection of things will permit.
"In the fall of 1839, I was much exposed, and laboured exceedingly
hard, which brought on an attack of fever, that seemed to spend its force
principally in my head. I also had a, severe cough, and at one time
spit blood. As the fever increased, I experienced a kind of stupor
and derangement of mind. In this state I had the most singular
dreams, or visions of things. One peculiar thought that entered my
mind was, that my body was divided into four parts; the legs being-
cut off at the knees, and my head and breast severed from the body,
which appeared to me to be real and true; and I suffered great
anxiety as to how the parts of my body should be re-united, and made
to grow together again. A physician Avas employed, and he ordered
plasters to be applied to my ancles, and a blister to my breast, and one
on the top of my head, and gave me several emetics; and the pain of
all these, and the distress of the fever in my head, was enough to render
the strongest man, with the best constitution in the world, senseless
and delirious.
" I continued in this condition some time, sometimes pretty sensible,
and at others indifferent to what presented itself before me. At length,
through the advice of some friends, I believe I Avas taken to your
asylum. As near as I can recollect, I Avas taken tAvice. The first time
there Avas no room for me, and my father had to take me home again.
I remember, on my first visit, of seeing the four round pillars in front
of the building, and of walking up the steps into your room. At this
time, I entertained the opinion of liaA'ing just landed in the city of
Eome; and, from the circumstance of noticing these pillars, and the
immense size of the building, I Avas induced to entertain the belief of
its being a house used by the Roman catholics for their religious
sendees. I thought it Avas a monastery. I also thought the piece of
ground, in front of the building, Avas holy and consecrated ground, used
by them for the interment of the dead. I suppose the reason Avliy
I thought so Avas, because the ground betAveen the gate and the house
had been fresh ploughed, and it looked yelloAV. I had an idea that the
LETTERS FROM PATIENTS. 473
Romans, and sonic other denominations, were exercising their autho-
rity upon young and old; and I thought I was brought here to
be scourged, and taken through purgatory. After I arrived the second
time, I thought that the building was used for a medical college, and
the inmates were going through a certain preparation, or process of
experiments, rendering them fit subjects for dissection and investi-
gation. After that, I concluded it was a kind of a fort for the protection
of the people of the country, for I expected that France had united
with the southern part of the United States, and we were suffering the
unpleasant consequences of a war. These, and a great many other
curious and singular notions, not necessary to mention, I entertained
through the winter and spring, and until I began to get better.
" My greatest trouble was, as to the place in which I was, and the
true use made of it. I made various inquiries of my companions {the
other patients) for correct information. I asked them often where I
was, but the answers which they gave induced me to disbelieve every
word they said; and it was a long time before I could credit anything
I was told. When I rcflect 011 the many incidents connected with my
sickness and recovery, I am amazed.
" In conclusion, I express my gratitude for the attention that you ren-
dered me, hoping that your skill and practice in the restoration of the
afflicted may be always attended with success; and, in the end, promote
the happiness and welfare of mankind, is the earnest desire of
" Your affectionate friend."
To Dr. Awl.
" Respected and dear Sir,?One year has passed away since I became
an inmate of that great and benevolent institution over which you
preside. When I compare my present condition with that at the com-
mencement of the year, then an object of pity and source of grief
to relatives and friends, but now in the full exercise of reason, and
in the enjoyment of bodily health, I feel that there is no one who
has more reason to be thankful to God and his fellow-beings than
myself.
" Lamentable, indeed, is the condition of one deprived of reason, and
taken from the sphere of usefulness in which he may have moved. But it
is pleasing to rcflect that our noble State, though still in her infancy,
lias erected so great an institution for the benefit of her unfortunate
children, where all the conveniences and necessary attentions for their
comfort are provided. Perhaps you wonder that I have not expressed
my gratitude long ere this, and, though I know I have been too
neglectful, still think it not yet too late. I have recently had the
pleasure of seeing two of my fellow-patients, Mr. 0. and Mr. C. I saw
the former at his residence, where he seemed to be deeply engaged in
business; was cheerful and happy, and as capable of attending to his
affairs as any other man. Mr. C. visited me a few days since, in health.
I was glad to see him, and hear from the asylum.
" But how am I to pay the debt of gratitude I owe to you, and those
in attendance, for the kindness and attention received while a patient
NO. XII. I I
474 RELIGIOUS INSANITY.
under your care ? If a place in memory would compensate, surely you
have it, for though seven months have gone since I left, a day does not
pass without my thinking of the asylum. And now please accept my
thanks for all the kindness and attentions received; and may you long-
live to see the fruits of your benevolent labours.
" With much respect and esteem, yours, <fcc.,
On the subject of insanity, associated with deluded religious notions,
the following judicious observations will be read with interest:?
"There is no country where the subject of religion is more imme-
diately and forcibly brought home to the heart and conscience, than in
the United States. It is one, too, upon which every variety of opinion
exists. Unlimited discussion is in constant practice among all classes;
and the feelings and apprehensions of our nature are much aroused and
frequently excited. And it cannot be doubted that in many cases
where persons are predisposed to this fearful disease, by natural consti-
tution, incorrect education, feeble health, and other circumstances,
anxieties, connected with the awful realities of eternity and the immor-
tality of the soul, have been the exciting causes of mental derangement.
But pure and undefiled religion, whose genial influences shed peace and
joy over the path of our existence, and light us with elevated hopes to
the prospects of a happy eternity, can, in its unperverted results, have
110 such injurious effects upon the mind. 'The caviler may accuse
religion of producing insanity, but lie docs not see how many causes of
insanity it averts, how much comfort it affords to the weary and heavy
laden, how effectually it buoys up the desponding, and how directly it
points to the transgressor the way of pardon and peace.'* As the result
of some attention to this matter, we feel satisfied that the true remote
cause of insanity very frequently lies behind the religious influences
which appear so conspicuous, that, at most, religion can only be accuscd
as the occasional or exciting cause of a disease whose foundation is
completely established in the system; that, in a great many of these
cases, the mental derangement will be found mainly to depend upon ill
health, or that peculiar debility and irritation of the nervous system
which so frequently follow various acute disorders that severely try the
organic structure, and, in not a few instances, so far is the disease of
the mind from a religious origin, that it is clcarly and properly charge-
able to the indulgence of vicious habits. It is certainly a fact that a
maniac may imbibe a religious as well as any other extravagant delusion,
and yet his derangement may be occasioncd by the very reverse of any-
thing like a religious cause. Some, indeed, never appear to speak
seriously upon the subject of religion, only when they are crazy, and
then it would seem as if the anguish of remorse had commenced a drill
upon the disturbed and distracted conscience.
" But the religious only appear to constitute the largest number of
exciting causes in our annual reports ; for if we carefully analyze the
tables, and faithfully consider those of intemperance, ill treatment,
* Dr. Woodward.
ORATION DELIVERED BY A LUNATIC. 475
domestic trouble, jealousy, masturbation, and, perhaps, several others,
with a fair proportion of the unknown, it will be found that vice pre-
dominates, and its victims far exceed all others."
It is customary, as most of our readers are aware, for the Americans
to celebrate annually the 4tli of July. This is one of their national
jubilees. Orations commemorative of the recognition of American
independence are delivered in nearly every town and village throughout
the country. It appears, however, that this practice is not confined to
the sane portion of the population, but that lunatics, confined in the
asylums, are permitted to give expression to the national feeling. The
following account of the celebration of the 4th of July in the Ohio
Lunatic Asylum, with the oration of . one of its inmates, cannot but
prove interesting to our readers, on more accounts than one. It con-
stitutes a psychological curiosity.
To celebrate the 4th of July, a large party of the inmates, of both
sexes, with the officers, attendants, and assistants of the asylum, assembled
at an early hour, in the third story of the west verandah, where they
were soon joined by the superintendent, teachers and pupils of the Ohio
Institution for the Instruction of the Blind, who kindly attended as
invited guests, bringing their best singers and all their instruments of
music.
The Declaration of Independence was read by Mr. J. S. T., of the
middle gallery, and an agreeable oration delivered by Mr. H. L. K., of
the first gallery, after which the company sat down to an excellent
dinner in the adjoining hall.
We append a copy of the oration for the gratification of those who
are curious in observing the peculiarities which mark a disordered mind.
" An Oration, delivered in the Ohio Lunatic Asylum,
July 4, 1846, or 1958.
BY H. L. K.
"Fellow Citizens :?With emotions of good will we salute you. On
this morn rose the seventieth sun of our national independence. It has
risen upon sixteen million of freemen generally in health. Once more
are we called upon to contemplate ourselves as a people ; to glance at
the situation of sister nations of the earth; and, amid a multiplicity of
favours, to be raised on the wing of devout emotion to the Supreme
Governor of the universe, whose goodness is unbounded, and whose wise
omnipotent finger touches all the springs of matter and of mind. We
are called upon, after the example of the wisest nations and patriots,
soberly (I do not say abstinently) to rejoice, and to express our joy in
becoming independent tones. What we propose at present, fellow
citizens, is, to lead the mind, not so much to one, as to many, grounds
of rejoicing.
" 1st. We enjoy the rigid of pursuing happiness according to what light
ii 2
476 ORATION DELIVERED BY A LUNATIC.
we have, which, next to health, is the greatest of all temporal blessings.
If dissatisfied with our situation in one section of the country, we are at
liberty to move either north or south, or east, or west; and we can find
friends wherever we go. We may turn our attention to mechanism, to
manufacturing, or to farming, as Ave choose. Or, if we wish to ascend
the hill of science, where is the literary taste called forth as in the
United States 1 The number of our universities, and colleges, and
seminaries, and schools, and periodicals answer the question. Profes-
sional men, of every description, with us receive patronage; and, not-
withstanding reflections from the more unenlightened, that the lawyer
only by degrees goes to heaven, it is an honour to be a member of the
bar. Do any wish to taste the pleasures of single life, and to taste
them all their lives, the fines imposed on celibacy, or bachelors, are not
generally large. Or do the bumps of amativencss and of philoprogeni-
tiveness appear prominent with many, a numerous family, even among
the poor, is one of the beauties of our country; and it is reputable to
get married either in the thirteenth 01* ninety-third year of our lives !
In some countries, parents enter into marriage contracts for their
children ; with us, young people make their own marriage contracts.
The daughter is not to inquire, mother, how long will it be before I am
old enough to get married '? It is generally believed she is old enough
to get married whenever she thinks she is ! In this we may err, and
doubtless do sometimes err, in not consulting parents and governors as
we should in the important concern of marriage. We sometimes hear
of flights by the light of the moon, but seldom a disappointed love-song.
" 2nd. Another source of joy is the enjoyment of the right of suffrage to
an extent unequalled. How many thousand fellow mortals are governed
by those in whose elevation over them they had 110 choice. It is not
so with us ; and grateful should we feel, this 4th of July, fellow citizens,
with sweet recollections of our entombed ancestors, because it is not so
with us. We have a choice in our officers, both civil and religious.
The question with us is not, how much honour or wealth have you ?
but it is, are you a friend to our government 1 in the years of maturity ?
if so, you must have a vote for every officer.
" 3rd. Our eligibility to office according to merit, is another source of
national joy. Whatever be our conscientious views of policy, and to
whatever height rises our political pulse, which is sometimes 120, and
which should, therefore, be reduced by gentle cathartics and diaphoretics,
if not by an emetic and venia section, it is joyful to know that we, as a
republican people, are all in love with our silver constitution; disposed,
on this day of joyful festivity, to unite as a band of brothers, to sacrifice
mere sectional feeling, in order to advance the meritorious, and to march
in unison against a common foe. The right of self-defence we take for
granted, because none ever hated his own flesh. We hail the European,
the Asiatic, and the African, and are disposed, as we should be, that
they, with us, participate in office. No sooner arc they under our
jurisdiction, as a united people, than they are free. We deny that the
United States' constitution is stained by a single blot of slavery. It
existed in some of the colonics before the adoption of the constitution,
ORATION DELIVERED BY A LUNATIC. 477
at which time the best of the circumstances was made ; and the best of
circumstances will still be made, in dutifully cleaving to it. To with-
draw from citizens, because they are disorderly, is to go out of the
world. In giving freedom, therefore, to all, even to 1 servants of ser-
vants.we should move temperately, knowing that the ministers of state,
to whom it belongs to sunder unrighteous chains, are ministers for good,
in the hands of high Heaven, beyond whose word and time of freedom
none can be detained in servitude; 110, not for an hour. With that
equality for which some contend we cannot go, an equality which would
annihilate the distinction between superiors and inferiors, and banish
from the world the idea of sovereignty. We cannot be persuaded that
white and black are one and the same.
" 4th. Another source of national rejoicing is, that benevolence and
beneficence are now flowing out of an irresponsible into the organized
channel of the powers that be. So far as civil, they are coming under
the immediate control of the nation or the States. The Unitarian
National Temperance Society will die of itself; as well as other irre-
sponsible societies, which are as numerous as the horns of the sea-
monstrous Apocalyptic beast. Beware (says Washington, in his fare-
well address) of societies which entrammel or overawe the constituted
authorities in deliberate movement. Behold the abolition, and the
multiplicity of the enthusiastic temperance petitions to strike at the
license system throughout the land, as if we should become a nation of
monks or of nuns ; or as if a twenty year gloomy spell of abstinence
from Rome, Mahomet, or the Devil, could always hold us from wine or
from women, from tea or from coffee, tartar-emetic or calomel, opium
or tobacco.
" As to the legislatures of the States as organized, it is pleasing to
know that they have not been deaf to the calls of the poor, of the blind,
the dumb, or the insane. Witness Massachusetts and Ohio with their
asylums. Of the 245 patients in this lunatic asylum, fellow-citizens,
110 doubt many do not feel as happy as they would; and many are
ready to attribute their misfortune to the legislature, or to Dr. Awl, ox-
others having immediate oversight. This, in general, is a great mistake,
arising from an erroneous idea. The legislature, physicians, and
attendants, are parents, servants, friends, to aid in removing or alleviat-
ing those distresses which they did not cause, but which a wise Pro-
vidence permitted. We are fallen creatures, and therefore liable to
diseased action. It may attack any part of our bodies, and it often
affects the nervous system; that mysterious organization of sensitiveness,
which conveys to the embodied mind the elements of thought! Because
she is nervous, idiotic, lunatic, or insane, we are not therefore always to
infer the Almighty has forsaken her. Many in the present life are
restored to good health, and many a bright soul, in the moment of dis-
solution, is doubtless wafted on angelic wing from a crazy, sickly
constitution, to regions of bliss eternal in heaven. Both Heman and
Timothy seem to have been nervous; and suppose ye that those
eighteen on whom the tower of Siloam fell, were sinners above all
others, because they suffered such things ? I tell you nay, says Jesus.
478 ORATION DELIVERED BY A LUNATIC.
Correction is promised to tlie good, and the casting out the dragon from
the church to the earth, and his pouring forth from his mouth Avater as
a flood to carry away the woman, and the help the earth is now giving
her by its asylums and laws to prevent mobocracy and persecution,
whilst the serpent is wroth, knowing that he has but a short time till
the .Millennium Sun, now risen, shines under the whole heavens, the
Lord God Omnipotent reignetli, are all matters of prediction at this
time literally fulfilling. In the mean time, while the distresses arc
such as never were since there was a nation, (I mean soul distresses,)
let us rejoice that Deity is, in the written word, the ground of faith
amid the storm, and that he is everywhere the object of admiration.
" Warms in the sun, refreshes in the breeze,
Glows in the stars, and blossoms in the trees," &c.
" To the rational complaining patient, however, we concede, that it
is often difficult to determine when many are insane; and that now
when the seventh angel is sounding, and truth has forsaken the earth,
an individual may be proceeded against, and committed by false tes-
timony to an asylum, where he should not be committed; or he may be
there detained when he should not be detained, or have his soul under
the altar. The responsibility of governors in this concern is truly
difficult and great.
" 5th. A fifth source of joy is liberty in dress. It is reputable with us
for a young man to appear in tights, or in pants wide as a pillow-case.
He will pass with seven dollars on his head, or with but twenty-five
cents. The rim of his hat may be an umbrella, or as if immediately
from Boston. A lady is loved in a cotton dress as well as in silk. If
she has a countenance indicative of some good quality, such as modesty,
sincerity, humility, or good sense, she appears well in a plain drooped
bonnet, or in a gipsy-hat; and she may wear it on her face, or 011 the
back of her head. She may either compress the arms below the
shoulders, or the ivaist; but the lacing of her feet in imitation of the
Chinese ladies will be less injurious to the health of her offspring !
Cossicks, or Gossicks, according to the German, arc suitable for mil-
liners and Aveavers. Upon the Avhole, as Ave can dress for church with
either^ve or fifty dollars, Ave enjoy much freedom from superstition.
" Gth. A sixth source of joy is the mildness of our government. I
deem it neither anti-national nor enthusiastic, AA'ith confidence to affirm
that Ave are the subject of prophetic story. ' I beheld another beast
coming up out of the earth, having tAvo horns like a lamb, and he spake
as a dragon.' One of the horns of this beast is the United States, the
mildness of whose government is lamb-like. John Adams, first vice-
president, and second president, emphatically, together Avith the other
ten presidents, being this beast, as he is civil; answering to the first
Apocalyptic beast Avith ten horns, Avhose seven heads, or mountains, or
churches of (instead of in) Asia, Avere thrust out from connexion Avith
the Nation by the Constitution, which declares that ' all power herein
granted shall be vested in Congress?which shall make no law to esta-
blish religion, nor to prevemt the free exercise thereof!'
ORATION DELIVERED BY A LUNATIC. 479
" 7tli. A seventh source of joy is, as a people we are tenacious of our
rights. The little clouds of contention now seeming to rise in our
horizon, we hope Avill be as the morning cloud. We wish to extend
our humane laws over our far west territory of Oregon; and to our
jurisprudence sister nations cannot object. Many of our laws we
borrowed from our neighbours, and, refined, we must give them to
the nations! Already are we honoured with foreign ambassadors, to
learn, and to imitate our prison discipline: and we mean that a sober,
steady, independent move shall characterize us,?always having the
generosity of the lion, which seldom devours animals that prostrate
themselves before it; spares women and children in preference to men,
and men rather than wild beasts.
" 8th. The last source of rejoicing, fellow-citizens, which we notice, is
the universal summons from the highest authority, to behold and hear us
?people. ' All ye inhabitants of the world,' says the seraphic Isaiah,
whose hallowed lips were touched with holy fire, and whose mind,
on the wing of sublimity, was now eight thousand miles beneath, in the
centre of the earth ; now in the third heaven?now round the universe
?now beyond the limits of time?far above all heavens?in the regions
of eternity,?1 all ye inhabitants of the world, and dwellers on the earth,
see ye when he (the United States) lifteth up an ensign upon the moun-
tains (in the adoption of the constitution), and when he bloweth the
trumpet (in the declaration of independence), hear ye? Isaiah xviii. 3.
This ensign, as it is spiritual, is now (1845 or 1958,) lifted up, in the
resurrection of the witnesses over the anti-cliristian kingdom, which
will be emphatically developed within forty-five years from this time.
But as it is a national ensign, it refers to our civil move upon the earth,
in order to enforce which, I close with the following resolutions:?
"At a meeting of citizens in the Presidential Chapel, as a Pope,
Ohio, July 4, 1846, it was unanimously
" 1. Resolved, That, in order to a constitutional, exclusive, and
untrammelled legislation in all cases whatsoever, the seat of General
Government be moved to the centre of the Union, in the neighbourhood
of Columbus, Ohio.
" 2. That all persons born in the United States, and in each of the
States, from and after January 1, 1845, are, in a civil sense, to all
intents and purposes, free.
" 3. That all male servants and slaves now in servitude, are free as
they arrive at the age of twenty-one years.
" 4. That all female servants and slaves now in servitude, are free at
the age of eighteen.
" 5. That the United States appropriate land, in the extensive north-
west, for forming a colony of blacks, as they become free, aside of their
half-brethren, the Indians.
" 6. That we, as a people, frown on privileged monopolies, and
voluntary irresponsible government in the midst of our government.
" 7. That, dropping party division, we unite in a National Consti-
tutional Bank, swallowing up the petty State unamenables!
" 8. That we exchange commodities with our brethren of the south?
480 ESCAPE OF PATIENTS PROM THE LUNATIC ASYLUM.
cast, and vest?keeping up our tariff: tliat is, our revenue and pro ?
tection; self-love being the rule of love ?political.
" 9 th. That Avithin forty-five years from 1844?that is, from 1957,
according to prophetic story,?the chains of slavery, of Home, and
Mahomet, shall have fallen; the Jew he in the Church; the saints noiv
having possession of the kingdom, will, in the character of citizen,
wield the sword under the whole heavens; the Avolf and the lamb shall
lie down together, and a little child shall lead them !
' This fourth July,
I'm three feet high;
I tell no lie,?
I'm born to (lie.
I'm not so tall,
Yet I am 1 All,'
At father's call,?
My mother's doll.
I love to see
Us people free,
And talk about
Our liberty.
Then let ns sing,
On Freedom's string,
A little cliild's
A nice plaything."
It is said that insane patients are incapable of acting in concert or
combination, and on this account they are more easily controlled; but
the following facts would seem to throw discredit upon the assumption.
However, a conspiracy like the one about to be recorded rarely, we are
happy to say, takes place in asylums for the insane. We never saw but
one instance in which two patients agreed to act conjointly, with the view
of escaping from an asylum:?
" F , Iv , M , and S , were inmates of the institution,
and members of the same class in the lower story of the building. The
first a Yankee, the second a German, the third an Englishman, and the
fourth a Pennsylvanian; but to make up the assortment, it happened
that the last was the son of a Scotchman. They were all comfortably
situated and doing well, especially the first three, who were considered
improving, and gave daily promise of favourable results. But becoming
uneasy and discontented, they began to consult together and contrive ways
of escape from the building, encouraged by the descendant of the Scot,
who had long been a troublesome fellow, and was frequently detected in
attempts to break out. At length a plan was proposed by the Yankee,
which met with general acceptance, for it was Ave 11 calculated to outwit
their friends, the doctor and his attendants, provided they could safely
elude the perpetual curiosity and vigilance of a very stirring gentleman
in the same class, whom they were afraid to trust, well knowing his
candour and disposition in such matters, and being fully apprised of his
partiality for the head of the institution, with whom he had made a very
satisfactory contract to study mcdicine for the period of twenty-one years.
But as this famous student was very fond of preaching, and could easily
be set a-going at that, it was proposed that one or two of the band should
keep him at this employment whilst the others were engaged in carrying
out their plan. Having procured the rusty blade of an old trowel, that
some one had carelessly left within reach, they commenced daily opera-
tions upon one of the front windows, and at last succeeded in removing
till the screws and other fastenings by which it was sccured, until it
SUDDEN RECOVERY FROM INSANITY. 481
could at any time be easily removed; carefully disposing of all dirt, and
filling up the screw holes with soft bread to prevent detection. All
things being ready for action, they selected an evening immediately
after the commencement of our religious services, as the best time to
take out the window, and give them all au opportunity to get out,
thinking it probable that their unsuspecting attendant would, upon that
occasion, accompany other patients and be a short time out of the way.
" Accordingly, when the time arrived, and the last stroke of the service
bell had fairly died away, and they had seen their attendant leave his
place, they began by mounting the student upon a chair at the opposite
end of the hall, with his back towards the unscrewed Avindow, and giving
him his favourite text; the iron sash Avas quickly removed Avliile the
preacher Avas in full swing, and each in succession commenced their
hasty escape. But' the best laid schemes o' mice and men gang aft
aglee.' It so happened that one of the ladies attached to the institution
Avas returning at that moment from church in the city; she gave the
alarm to an attendant in sight, but only in season to secure the unlucky
Scot, just as he Avas reaching the ground in jumping from the AvindoAv.
The others had got doAvn before him, and, taking to their heels, Avere
soon out of sight in the neighbouring Avood. Every hand that could be
spared from duty immediately started in pursuit, and it Avas but a short
time before a faithful and actiA'e attendant, Avell up to business of
this nature, got upon their route, and succeeded in taking the Avliole of
them together, at the distance of tAveh'e miles from the asylum. He
brought them all back in a farmer's Avagon, hired for the purpose.
" They Avere kindly receiATed, and returned to their old quarters, Avliere
in due time Ave had the satisfaction to restore the German and the
Englishman fully to their reason. The Yankee aftenvards broke out
again and ran oft', but he Avas so nearly Avell that he arrived at home safe
and in the possession of his reason. The fourth being incurable is still
in our care, and nearly as troublesome as ever. The student likewise
remains. He is still satisfied Avith his contract of tAventy-one years, and
pleased Avith the prospect of getting through his studies at the end of
that period; after which he thinks it not unlikely he will take a full
course on diA'inity, if the doctor has no objection."
The folloAAring case is illustrative of that sudden restoration to reason
Avhich AAre occasionally Avitness among the insane, and Avliich bids us
never despair, even when but a feAv rays of intellect cast their cheering-
influence over the darkened understanding !
"The patient Avas a pleasant little Avoman, of delicate make, and
rather feeble constitution. The Avife of a young farmer, in this State,
just commencing life, Avliose slender resources AArere quite exhausted in
proATiding for, and taking care of her, during her sickness and insanity.
Her derangement Avas caused by puerperal compulsions, and at the time
of admission, had continued betAveen fiAre and six months, AArithout a
lucid interval. When recei\*ed, she Avas noisy, incoherent, and careless
of her habits and personal appearance, and A'ery much emaciated and
7
482 ADVANTAGE OF FARMING LABOUR TO THE INSANE.
reduced in strength. So wretched was her condition, and so few the
remaining traces of intelligence in her poor thin little face, that for a
long period, her case was regarded as utterly hopeless and lost. For
weeks she continued talking, and muttering to herself, in the most
imbecile and childish manner, with very little intermission, cither night
or day, frequently lying down upon the floor, or sitting in some retired
corner of the building for hours together. Every effort in our power
was made for her personal comfort and relief, by a properly-regulated
diet, and such medicines as were suitable; but it was a long period
before there was any visible token of amendment, or encouraging cir-
cumstance to give us hope. At length her scattered senses, and
bewildered mind, seemed to be less confounded, her appetite improved,
and she began to inquire a little, and show some degree of interest in
surrounding objects ; and then to request employment, which was given
with the happiest effect.
"But still her mind continued weak, and frequently disposed to
wander, and there seemed to be the greatest difficulty in regard to her
personal identity. She for several Weeks believed herself to be a horse,
or cat, or some strange animal. One day, however, she suddenly came
to herself in a manner equally simple and surprising. She Avas quietly
engaged with her needle, and after looking steadily for some time at
her hand, she all at once exclaimed?'Well, now do see, if there ain't
that same little old scar behind my thumb, and now I know it's me,
sure enough !' From this time forward, every trouble and delusive
feeling entirely vanished from her mind, and we had the unspeakable
pleasure of seeing her perfectly restored to the enjoyment of reason and
health,?a well-behaved, industrious, and excellent woman, fully sen-
sible of the great change effected in her condition, and very grateful for
our services, and the kind treatment she had received at the institu-
tion."
The Report speaks highly of farming and gardening as being useful
and pleasing to the insane. They afford just the kind of employment
which they can profitably follow, both for themselves and for the
institution in which they are confined. Amongst many other requisites,
the patients have this great advantage over everything else, that all
classes of males, from the highest to the lowest, who arc in a condition
to labour, can in some degree be thus employed. Many of the patients
are very judicious farmers, and the light-minded and loquacious imbecilc
can learn to dig and plant with very good effect; even the poor snail-
like mortal whose mind is nearly lost in a state of dementia, can find
something of interest or pleasure to engage him when allowed to be
with others who are at work in the field.
By useful labour the weary hours of confinement can almost daily be
relieved. Exercise in the open air not only invigorates the frame, but
by presenting new objects, changes the feelings, and removes distressing
thoughts. Refreshing sleep will follow daily toil?physical health is
certain to be increased, and the disordered mind will rarely fail to be
THE PATHOLOGY OF INSANITY. 483
improved. The mind is, indeed, in this way, not unfrequently revived
from the most listless and hopeless condition.
Nor are the benefits of the farming lands confined to the men alone.
The female patients are also fond of cultivated fields and shady groves.
They love to take their pleasant walks through well-made gardens, and
along winding paths, enjoying the balmy air and cheering sun. We
append a copy of some verses composed by one of the patients upon a
little bud of spring-flower, which reared its solitary head, and graced the
window of the patients' sitting-room. It is addressed to the " Rose-bud
in the Window?
" I have often seen the flower spring
From out the mould'ring wall?
I have seen the clust'ring blossoms cling
And grace the ruin'd hall.
But Lere, 'mid scenes of human woe,
This little rose intends to blow.
So in life's shades, however drear,
Some raj- of mercy will appear.
Bloom, tiny flower, a gracious hand
Invisible, unfolds tby leaves
O'er scenes of grief, by liis cotmnand
Joy still with sorrow interweaves.?Charlotte.'"
The following practical observations 011 the use of depleting remedies
in the treatment of insanity, are deserving of attention and of record:?
" I cannot, however, permit this opportunity to pass, without a Avord
or two to my professional brethren of the West, upon the effects of
extensive depletion in the treatment of this disease, which I shall briefly
submit, in the most respectful manner.
" The pathological condition of insanity, in its primary and active
stages, appears to be one of peculiar irritation, and not of the ordi-
nary character of inflammation; and, by common consent, it is now
settled, to the satisfaction of a large majority, if not all, the medical
superintendents in the hospitals of the United States, that much general
depletion, particularly by means of the lancet, in acute mania, (the only
form of the disease in which it is likely to occur,) is very generally pro-
ductive of injurious rather than beneficial effects. That the symptoms
which seem to indicate the use of blood-letting so strongly, are decep-
tive, exhibiting to the practitioner the effect, and not the cause, of
excitement. And Avliile active and excessive depletion may rapidly
sink the physical strength, it at the same time renders the nervous
system more susceptible and irritable, the actual violence of the disorder
not only remaining unsubdued, but often is thereby increased. It is
known that the loss of large quantities of blood is frequently succeeded
by severe pain in the head, as if it were surrounded by a tight band, or
having the sense of an iron finger pressing upon some particular point
of its surface; together with ringing in the ears, rushing and drawing,
or cracking noises; vertigo, wakefulness, or starting during sleep;
484 MEDICAL TREATMENT OF INSANITY.
intolerance of light and sound, and many strange sensations and illusive
feelings, which tend to permanent lunacy, or are productive of an alarm-
ing state of anemia and chaos of intellect, from which weeks and months
of care and judicious treatment can scarcely redeem the patient. These
symptoms are, in some degree, calculated to mislead, and, as this state
of exhaustion may follow what, in other circumstances, would justly be-
considered moderate and useful bleeding, there is the greater need for
caution. ?
" I am a great friend to the lancet. An experience of twenty years,
in general practice, has fully satisfied my mind that it is one of the
greatest and best remedies we possess in the treatment of many acute
diseases. But observation and reflection have also demonstrated that
its frequent use is both unsafe and injudicious in the treatment of
insanity.
" When the patient is young, vigorous, and athletic, has redness of the
eyes, and complains of much headache, and there is, at the same time,
considerable heat about the head, with throbbing of the carotid and
temporal arteries, a single bleeding may not be incorrect; but even
here, it is probable, local bleeding, by means of cups or leeches, Avitlx
the constant application of cold water to the head, would be sufficient
for every useful purpose, and, on several accounts, is greatly to be pre-
ferred.
" The frequent use of drastic cathartics is also objectionable, on
account of the danger of producing irritation in the bowels, which may
lead to diarrhoea or dysentery?troublesome conditions of disease, to
Avhich the insane are extremely obnoxious. Costiveness is undoubtedly
to be obviated; but, for this purpose, the milder laxatives will generally
succeed the best. Blue pill, and small doses of calomel, will be found
useful, as in other cases, where the sccretions of the liver arc unhealthy
or deficient.
" Before closing this report, it seems, necessary that I should repeat a
caution which was formerly given, in reference to the practice of making
false promises, or using deception of any kind, in order to induce insane
persons to leave home and come to the Asylum. The effects of this
practice are often extremely unpleasant and trying to their feelings. It
is also very likely to excite suspicion and prejudice against the officers
and attendants in the institution, whom they naturally take to be parties
in the supposed scheme of their unlawful arrest, and unnecessary deten-
tion, all of which has a tendency to lessen our influence, and perhaps
prevent the success of a proper course of treatment. We have had
a^ number of melancholy examples of bitter feelings, and strong aver-
sions to parents and friends, thus produced, which neither time nor
attention could wholly remove."
Much discrepancy of opinion exists as to the influence of the different
seasons in inducing insanity. To those who are interested in this
inquiry, the subjoined table will prove valuable.
INFLUENCE OF THE SEASONS ON THE INSANE. 485
Statistics of Different Seasons.
Admissions in winter .
Admissions in spring .
Admissions in summer
Admissions in autumn
Discharges in winter .
Discbarges in spring .
Discbarges in summer
Discbarges in autumn
Recoveries in winter .
Recoveries in spring .
Recoveries in summer
Recoveries in autumn
Deaths in winter . . .
Deaths in spring . . .
Deaths in summer . .
Deaths in autumn . .
1839. 1840. 1841. 1842. 1843. 1844.! 1845. 1846. 1847. Totals.
45
21
59
32
4
4
5
20
1
4
0
19
3
0
>)?)
225
257
338
149
107
183
219
70
100
117
101
34
20
32
30
It appears from tlie statistical records of tlie asylum, tliat tlie number
of recoveries liave been most numerous in autumn, and less in winter,
which corresponds, we believe, with observations elsewhere. There have
been most frequent admissions in autumn and summer; but this is in
part, if not wholly, explained by the opening of the new buildings,
which, for two successive years, has taken place in the fall?also, by the
room afforded in consequence of more frequent recoveries at these
periods. Mania has been supposed to prevail most in summer, melan-
choly in autumn, and dementia in winter. It is certain that a conti-
nuance of warm weather augments excitement, while cold prolongs
depression. Some individuals pass the summer in a state of entire
freedom from all excitement, whilst in winter they are in an opposite
condition. Others are excited at irregular periods, both as to time and
season, and in a majority perhaps, especially in females, the exacerba-
tions occur monthly. Summer is thought to be most favourable to the
cure of dementia, and relapses are said to occur most readily at the
season of the year corresponding to the attack.
With regard to lunar influences, the question is asked, Docs the moon
exert any special influence upon the insane 1 The Germans and Italians
believe it does. Esquirol says : " I have been unable to verify this
influence, though I have been at some pains to assure myself of it.
It is true that the insane are more agitated at the full of the moon, as
they are also at early dawn. But is it not the light of the moon that
lit!//
480 INFLUENCE OF THE MOON ON THE INSANE.
excites tliem, as that of the day in the morning V Dr. Burrows remarks,
that, "the most accurate observation for many years, in extensive
communities of insane persons, contradicts the influence of the phases
of this planet on the human mind;" and Dr. Bush was of opinion "that
the cases are few in which mad people feel the influence of the moon;
and that when they do, it is derived chiefly from an increase of its
light." Similar observations are made by others having an opportunity
to notice the insane.
Dr. S. B. Woodward, late superintendent of the State Hospital, at
Worcester, Massachusetts, at the suggestion of one of the most scientific
men in New England, commenced a table of observations on the
influence of this planet upon the paroxysms and deaths of the insane,
and after much time devoted to the subject, says:?"These facts and
coincidences we leave for the present, with the single remark, that no
theory seems to be supported by them, which has existed cither among
the ignorant or the wise men who have been believers in the influence
of the moon upon the insane."
'jSxA ' The Report continues,? J- r> > ' Y
" To this highly respectable testimony we may add our own limited
observation, which must incline us to agree with what has been said.
Many patients are certainly more excitable and restless in pleasant
moonlight nights than in dark and gloomy weather; but this would
seem to be occasioned by the real or imaginary sight of objects in or
without the building, such as men, trees, animals, &c., or the motion,
perhaps, of the passing clouds. An opinion, however, that has existed
for so long a period, which has spread so extensively, and which, in
this country, is familiar as ' household words,' deserves to be carefully
examined; for it is important to disprove error, as well as to establish
truth.
" Weak and timid females arc sometimes alarmed and much agitated
during the continuance of lightning and thunder; but, as a general
thing, we have not observed the insane to be much disturbed on such
occasions."
The following tables will be read with interest:?
Classification.
Species of Insanity.
183Q. 1840. 1811. 1842. 1843. 1844. 1845. 184G. 1847. Totals
Mania
? epileptic variety . .
? homicidal variety
Melancholia
? suicidal variety
Moral insanity
Dementia
Idiotisin or imbecility . .
102
12
4
17
4
5
10
3
107
10
1
12
7
1
12
137
0
3
14
11
1
3
110
10
4
l(i
29
"o
712
ft 7
14
112
82
13
04
3
L
DEBATE AMONG THE LUNATICS OF THE ASYLUM. 487
Classification in reference to recoveries in each variety of insanity
discharged in nine years.
1. Mania . .
Males
Females.
2. Melancholy
Males
Females.
3. Demency .
Males
Females .
4. Epilepsy .
Males
Females.
Totals
Whole No.
discharged.
509
108
G4
37
718
No. of cach
sex cured.
210
147
35
38
0
10
Total of
cures.
357
73
15
3
448
Per centum of
recoveries in
each variety.
70-12
G7-50
23-59
8-J0
We have previously directed tlie attention of the curious to an oration
delivered by a lunatic patient in this Asylum on the 4th of July; but
what will our readers say to a debate on a point of philosophy, science,
and morals, carried on by insane persons confined in an asylum ? We
have heard of the parties, the balls, the friendly reunions, and even of
theatrical performances in our English institutions; but we never before
heard of a serious, a grave, and learned discussion, originating with, and
carried on by the patients, after having formed themselves into debating
societies! From the Ninth Report of the Ohio Lunatic Asylum, we
copy the following extract:?
"THE DEBATE.
" Having for some time noticed a strong disposition in two gentlemen
occupying the same gallery, to discuss certain topics of a general
character, especially the customary use of tobacco and ardent spirits, we
at length proposed that all strife should be ended by a public debate,
for which each party should have due time to make preparation. To
this there was an agreement, after some diplomatic finessing on both
sides. The day was accordingly fixed, and the question finally deter-
mined in writing, as follows:?1 Is it essential to the happiness and
comfort of man, tliat the use of tobacco and of ardent spirits be continued V
It was also arranged that a president, and two vice-presidents, and
a secretary should be chosen?that reporters should be admitted, if
desired, and that each debater should speak, if he thought proper, to
the extent of ten minutes by the president's watch, and no longer.
" Accordingly, at the appointed time, due preparation was made in
the gallery occupied by the gentlemen aforesaid, by the arrangement of
488 DEBATE AMONG THE LUNATICS OF THE ASYLUM.
seats for the officers of the occasion; and the disputants having signified
their readiness to begin the discussion, the superintendent of the
Asylum had the honour to he chosen president, assisted by two patients,
avIio were nominated and duly elected vice-presidents. A secretary was
.?also selected, and the assistant physicians proffered their services in the
capacity of reporters.
" The gentleman in the affirmative made his appearance with great
confidence, being well supplied with an immense roll of written matter,
the production of which had occupied much of his time for several days.
His argument began at the garden in Eden, where he was confident
tobacco, corn, and rye, were amongst the other goodly plants that grew
therein. ' That every plant was made for the use of man, and the only
modes of using tobacco, yet discovered, were to chew, smoke, or snufi' it.'
'That viewed in the glass of nature and judgment of the earth, the sober
use of tobacco and spirits were promoters of happiness and comfort.' Ap-
pealed to man at the first?to man after the flood?to man in the days of
Aliasuerus?to man in the times of the Gospel, and since the Declara-
tion of Independence, alleging that these things were nowhere strictly
forbidden?but were allowed by the church, and that tobacco was freely
used by ministers of all denominations. Attempted to show the value
of exports of tobacco, and the amount paid on foreign importations of
wine, spirits, gin, etc. Contended strongly that what had been said
against the use of good liquor, was addressed to the animal feelings,
in the recital of miserable anecdotes and impudent allusions to ' a
drunken breath, and weeping wife? And believing this opposition was
iin infringement of personal liberty, he was proceeding to show that
' the earth is now under anathema for cursing good creatures,' when the
president's hammer came down upon the table, the time under the rule
having expired, and lie very pleasantly took his seat.
" His opponent rose with great mildness, but in a very dignified
manner, remarking modestly, that lie thought ' if the company, and
especially the speakers, were blessed with small quantities of the matters
in question, it might serve to give spirit to the debate.' The gentleman
in the affirmative was particularly gratified with this remark, and seemed
to hail it as a yielding of the point. This, however, was but the glance
of a sunbeam before the refreshing shower, for he soon proved to be
anything but a novice in debate, opening the discussion with consider-
able ability, and meeting the arguments of his antagonist with great
readiness and skill?showing that they were light and false?that
tobacco was an injury to the system, no matter where it grew, and not-
withstanding its use by different clergymen?that ardent spirits were a
curse, and the cause of all unlmppiness in society?c that those families
were unquestionably the purest where the least liquor ran in the blood
and that the voice of truth and experience were against the use of these
articles in any shape. c Mr. President,' said lie, (if the gentleman is
right?if it be a fact that tobacco and strong drink are of any benefit in
society, I sir, for one, have lost my reason.' Continuing the argument
in good style, he was about to conclude in the most triumphant manner,
when some unfortunate whim in the brain gave his thoughts a new
STATISTICS OF AMERICAN HOSPITALS. 489
turn, and he took off in an opposite direction with the greatest rapidity,
arguing and flourishing away in the most lively manner, until, to the
astonishment of all, he was fairly on the ground that should have been
occupied by his adversary. Discovering the mistake, he made an effort
to recover, but had not time before the president's hammer was heard
upon the table.
" Reply, explanation, and rejoinder followed, but the above contains
the substance of the main discussion. Both gentlemen are regarded as
incurable maniacs, and it is curious that the order of debate was strictly
observed, nor did any evidence of ill humour appear.
"All parties now looked to the president for his decision. This
appeared to be rather non-committal. And as each party appeared to
be dissatisfied with the limited time allowed for speaking, and were
anxious for a second trial, he was about to postpone his final judg-
ment on the respective merits of the gentlemen in question, and
appoint a second meeting, when one of the vice-presidents suddenly
exclaimed?c Dish court ish all for no rise?any fool midout no sense at
all might knoio dhrinkin too much wasn't good for nobody /' Whereupon
the meeting adjourned sine die."
*******
" The following tables show the annual expenses of supporting hos-
pitals for the insane in America and in Great Britain. In making the
list, the writer of the Report has selected from the documents of the
State institutions alone. The private or corporate hospitals are probably
more expensive.
American Hospitals.
Institutions.
Maine at Augusta.
N. H. at Concord .
Yt. at Brattleboro'
Mass. at Worcester
Ct. at Hartford
N. Y. at Utica . .
Va. at Williamsburg
Va. at Staunton .
S. C. at Columbia.
Ivy. at Lexington .
Oliio at Columbus
184G
1847
1847
1847
1847
1847
1840
184(5
1847
1847
1847
Number of in-
mates during
the year.
187
187
420
G07
227
802
100
274
103
311
472
Average
number.
94
377
127
245
318
No. at the
close of the
year.
100
100
291
394
US
472
140
217
74
247
329
Current
Expenses.
?13,530 32
10,218 GO
20,445 80
37,090 05
20,894 73
47,985 45
21,300 23
29,040 13
11,728 02
20,080 74
28,070 21
NO. XII.
K K
490 MIXED INSANITY?REASON AND MADNESS.
British Hospitals.
Names.
Years.
Number of
residents.
Average.
Expenses.
Expenses.
Eetreat, York
St. Lake's
York Asylum
Cornwall.
Leicester.
Staffordshire
Kent . .
Dorset
Hanwell .
Edinburgh
Belfast
Carlo w
Lincoln .
Lancaster
Dundee .
Glasgow .
1843
1842
1842
1842
1841
1842
1842
1842
1842
1842
1842
1842
J 842
1830
1836
1842
112
457
189
J 00
352
100
300
107
30!)
89?
102
100
240
200
105
943
00
248
102
100
305
129
19G
?4,924 3 8
7,518 0 3
5,020 17 3
2,081 G 9
2,001 3 0
5,091 15 9
4,439 7 3
1,978 10 0
21,990 2 1
1,894 ]4 7
3,702 4 4
2,532 15 9
4,599 9 9
4,492 5 7
2,045 5 11
5,290 0 7
45*23,832 00
30,383 84
27,233 91
12,977 07
12,589 50
27,548 25
21,480 50
9,025 79
100,052 50
9,180 03
18,209 13
12,284 02
22,307 40
21,752 03
12,803 23
25,050 04
With these interesting extracts we are compelled for the present to
conclude our running commentary of these deeply interesting and
valuable reports. We cannot, however, do so without acknowledging
the gratification we have derived from their perusal, and expressing our
obligations and thanks to Dr. R. C. Hopkins for affording us this oppor-
tunity of bringing before the British psychologist the facts contained
in these records of one of the great American national state institutions
for the treatment of the insane.

				

